# The lifelong socioeconomic disadvantage of single-mother background - the Helsinki Birth Cohort study 1934–1944

**DOI:** 10.1186/s12889-016-3485-z

**Published:** 2016-08-18

**Authors:** H. Maiju Mikkonen, Minna K. Salonen, Antti Häkkinen, Maarit Olkkola, Anu-Katriina Pesonen, Katri Räikkönen, Clive Osmond, Johan G. Eriksson, Eero Kajantie

**Affiliations:** 1 National Institute for Health and Welfare, P.O. Box 30, FI-00271 Helsinki, Finland; 2 Faculty of Medicine, University of Helsinki, Helsinki, Finland; 3 Folkhälsan Research Centre, Helsinki, Finland; 4 Department of Political and Economic Studies, University of Helsinki, Helsinki, Finland; 5 Institute of Behavioural Sciences, University of Helsinki, Helsinki, Finland; 6 MRC Lifecourse Epidemiology Unit, University of Southampton, Southampton, United Kingdom; 7 Institute of Clinical Medicine, University of Helsinki, Helsinki, Finland; 8 Unit of General Practice, Helsinki University Central Hospital, Helsinki, Finland; 9 Vaasa Central Hospital, Vaasa, Finland; 10 Children’s Hospital, Helsinki University Central Hospital and University of Helsinki, Helsinki, Finland; 11 PEDEGO Research Unit, MRC Oulu, Oulu University Hospital and University of Oulu, Oulu, Finland

**Keywords:** Lifecourse, Marital status, Out of wedlock birth, Single parent, Single mother, Socioeconomic position, Unmarried mother

## Abstract

**Background:**

Growing up with one parent is associated with economic hardship and health disadvantages, but there is limited evidence of its lifetime consequences. We examined whether being born to an unmarried mother is associated with socioeconomic position and marital history over the lifespan.

**Methods:**

We analysed data from the Helsinki Birth Cohort Study including birth, child welfare clinic and school healthcare records from people born in Helsinki, Finland, between 1934 and 1944. Using a unique personal identification number, we linked these data to information on adult socioeconomic position from census data at 5-year intervals between 1970 and 2000, obtained from Statistics Finland.

**Results:**

Compared to children of married mothers, children of unmarried mothers were more likely to have lower educational attainment and occupational status (odds ratio for basic vs. tertiary education 3.40; 95 % confidence interval 2.17 to 5.20; for lowest vs. highest occupational category 2.75; 1.92 to 3.95). They were also less likely to reach the highest income third in adulthood and more likely to stay unmarried themselves. The associations were also present when adjusted for childhood socioeconomic position.

**Conclusion:**

Being born to an unmarried mother, in a society where marriage is the norm, is associated with socioeconomic disadvantage throughout life, over and above the disadvantage associated with childhood family occupational status. This disadvantage may in part mediate the association between low childhood socioeconomic position and health in later life.

## Background

Growing up with one parent has become increasingly common for children in the Western world. From 1980s to 2008 the share of single parent families in Europe rose from 10 to 21 % mainly due cohabitations and divorces but there are demographic differences in single parenthood between European countries [1]. However, in Europe and elsewhere low socioeconomic position and poverty is more common in single parent households, especially those headed by a woman [2, 3].

The lifelong consequences of low childhood socioeconomic position (SEP) has been extensively studied [4, 5]. In most studies parents’ low occupational status has been used as an indicator of childhood SEP [6, 7], while markers indicating other aspects of potential early life deprivation, such as mother’s marital status, have been less frequently used. However, numerous studies show that single parent family background is highly relevant determinant of health at least among children and young adults, among whom it is associated with adverse physical and mental health outcomes [8–12] as well as poorer educational performance and idleness (being neither in school nor employed) [11–14]. Less is known about the effects of single parent background across the life course, because most of the previous studies are performed in relatively young cohorts. Those few studies that have focused on lifelong effects of single motherhood have indicated that at least men born outside of marriage have poorer health and higher mortality and they are more likely to stay unmarried or become divorced [15–18]. Some studies also suggest that people born out of wedlock attain lower adult socioeconomic position (SEP) than those born to a married mother [16, 17]. However, it is not clear how the lifelong consequences of being born to an unmarried mother are mediated through the other childhood socioeconomic factors, and results in these mediating associations are contradictory [10, 12, 19–23].

Further, in many studies single parent families are also seen as a homogenous group, although it includes children who grow up with a single parent from birth to adult life, and children born to an unmarried mother who later marries and the child grows up with two parents or caregivers. Less attention has been paid to outcomes among different subgroups such as children born out of wedlock. Thus, a recent review concluded that research has not distinguished out of wedlock birth from single parenthood in general and called studies focusing on outcomes in different subgroups [13].

The main objective of this study was to examine whether being born out of wedlock in a society where marriage was the norm is associated with socioeconomic position and marital status in later life. The secondary objectives were to assess whether these associations are mediated through perinatal and childhood factors associated with being born to an unmarried mother and whether they are specific to children with no indication of a male caregiver rather than those who later had a male caregiver. In measuring socioeconomic position in adulthood we used different indicators of SEP from several time points across adult life.

## Methods

This study is a part of the Helsinki Birth Cohort Study (HBCS), which includes 13345 people born in either of the two public maternity hospitals from 1934 to 1944. The HBCS has been described in detail [24] and has been approved by the Ethics Committee of the National Public Health Institute. Register data were linked with permissions from Statistics Finland and the Finnish Ministry of Social Affairs and Health. Early life data came from birth, child welfare clinic and school healthcare records.

### Mother’s marital status

**Fig. 1:**
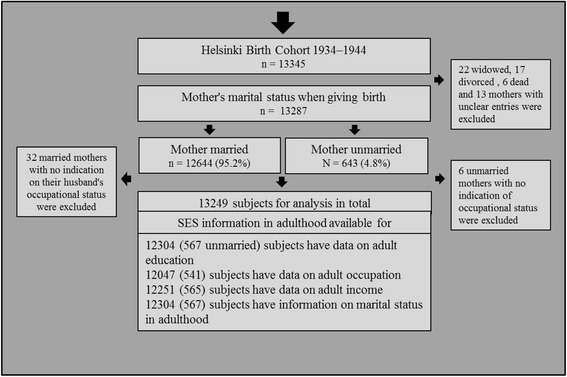
Flow chart of the sub-groups of the HBCS participants included in the present study

We defined mother’s marital status, as married or unmarried, when giving birth. The subgroups of the study are illustrated in the Fig. 1. Most married mothers (*n* = 12635) were classified based on information on the mother’s current last name and maiden name, as at that time women were by law required to adopt their husband’s last name when married. They also had an entry at least in one of the following sections: mother’s occupation when married, father’s occupation or father’s name. In the married group we also included 9 women who had a different last name in the birth record but who had no ‘unmarried’ entry and had obtained their husband’s name according to other records.

Most unmarried mothers (*n* = 531) were identified based on an entry ‘unmarried’ in the birth record. 107 mothers had no entry in the marital status section and five mothers had unclear entries in the name sections but neither group had any indication of the child’s father in the records. Thus, they were identified as unmarried. In addition, 22 women were widows and 17 divorced according to the birth record. Six mothers had died either in delivery or later according to the child welfare clinic or school records, and in 13 further cases we were unable to determine the mother’s marital status because of unclear entries. These 58 cases (0.4 %) were excluded from the analyses. Furthermore, we also excluded 32 married mothers with no information on the male caregiver’s occupation and 6 unmarried mothers with no information on the mother’s occupation. In our final analyses we included 6921 (52,2 %) male and 6328 (47,8 %) female offspring, in total 13249 people.

We have no data that would systematically indicate whether the parents were divorced or the father died later on. In a subset of 1658 subjects of the cohort, 28 (1.7 %) indicated that their father had died in the war [25]. This number is small and death from other reasons and divorce were rare at that time. Therefore, we have included these subjects in the analysis.

### Historical context

In the interwar period in Finland almost 10 % of babies were born ”illegitimate”, as they were referred to at that time. At the same time unmarried motherhood became more problematic when paid work outside home became more common for women and moral values underlined family values. Unmarried mothers were usually women from lower social classes [26]. The 1934–1944 period, during which our cohort was born, represents a period of transition in Helsinki. Philanthropically organized child welfare clinics offered guidance in child care and child welfare developed, in part to combat the increased mortality of “illegitimate” children. However, this time period also includes World War II, during which Finland fought two wars against the Soviet Union.

### Other variables

The HBCS also includes information on living conditions in childhood. As descriptive characteristics, we present the number of rooms and people living in the same childhood household (Table 1), obtained from the child welfare clinic record. As a covariate in our analyses we used information on 1770 children (13.4 %) who were evacuated unaccompanied by their parents to temporary foster homes in Sweden or Denmark during World War II. These children have poorer health and lower SEP in adult life [27, 28].

**Table 1:** Maternal and neonatal characteristics of the study participants (*n* = 13249) according to mother’s marital status

	Married	Unmarried
	Mean (SD)/n (%)	Mean (SD)/n (%)
	*n* = 12612	*n* = 637
Mother		
Height (m)	159.9 (5.6)	159.1 (6.5)
Weight (kg)	67.1 (8.3)	65.5 (8.1)
Body mass index (kg/m^2^)	26.2 (2.9)	25.9 (2.6)
Age (years)	28.5 (5.4)	26.5 (5.8)
Neonate		
Birth weight (g)	3413.2 (478.4)	3274.7 (465.6)
Head circumference (cm)	35.0 (1.5)	34.7 (1.5)
Birth length (cm)	50.3 (1.9)	49.8 (2.0)
Ponderal index (kg/m^3^)	26.8 (2.2)	26.5 (2.5)
Gestational age (days)	279.6 (12.9)	277.0 (16.7)
Birth order, first born	5929 (47.0 %)	495 (77.7 %)
Paternal occupation^a^ (highest) *n* = 12946	*n* = 12612	*n* = 334
Senior clerical	2170 (17.2 %)	53 (15.9 %)
Junior clerical	3073 (24.4 %)	64 (19.2 %)
Worker	7369 (58.4 %)	217 (65.0 %)
Maternal occupation (highest) *n* = 11819	*n* = 11182	*n* = 637
Senior clerical	484 (4.3 %)	13 (2.0 %)
Junior clerical	4363 (39.0 %)	141 (22.1 %)
Worker	6339 (56.7 %)	483 (75.8 %)
Rooms in childhood household *n* = 9383	*n* = 8927	*n* = 456
1 room	3530 (39.5 %)	266 (58.3 %)
2 rooms	3938 (44.1 %)	146 (32.0 %)
3 rooms	1124 (12.6 %)	37 (8.1 %)
4 or more rooms	335 (3.8 %)	7 (1.5 %)
Habitants in childhood household *n* = 8321	*n* = 7933	*n* = 388
2 habitants	179 (2.3 %)	58 (14.9 %)
3 habitants	3448 (43.4 %)	149 (38.3 %)
4 habitants	2519 (31.7 %)	97 (24.9 %)
5 or more habitants	1787 (22.5 %)	84 (21.6 %)
Temporary evacuation abroad to a foster family during World War II (*n* = 13062)	1671 (13.4 %)	99 (15.8 %)

^a^Father or other male caregiver

### Early life socioeconomic position

We defined childhood SEP based on the mother’s and father’s (or other male caregiver’s) highest occupational status as indicated in birth, childhood welfare clinic and school records. We classified the mothers’ occupational information according to the 1980 Classification of Occupations by Statistics Finland [29]. We used a 9-level socioeconomic coding (1 = employers, 2 = self-employed, 3 = senior clericals, 4 = junior clericals, 5 = manual workers, 6 = pensioners, 7 = students and pupils, 9 = unknown occupation/information missing). There were no pensioners among the subjects, and values indicating students and pupils and unknown occupations were set as missing. Using the highest category of the four time points (occupation before and during marriage in birth records, and current occupation in child welfare clinic and school records), we divided occupations into two categories: middle class (categories 1 to 4) and workers (category 5). Occupational information was available for 11819 mothers (89.2 %) (Table 1). Male caregivers’ occupational information was available for 12946 of the male caregivers and has been classified earlier using the same classification (97.7 %) [27].

We combined male caregiver’s prospective occupational information with mother’s marital status by dummy variables indicating both categories, with mothers married to manual worker male caregivers as a reference category. We performed these analyses in two ways: not adjusting and adjusting for mother’s occupational status (worker/middle class).

### Socioeconomic position in late life

Using a personal identification number, we linked early life data to information on adult educational attainment, occupational status, income and marital status, obtained from Statistics Finland at 5-year intervals between 1970 and 2000. We grouped maximum achieved occupation into four categories (1 = manual workers; 2 = self-employed; 3 = low official; 4 = high official) [30]. Education was grouped into four categories (1 = Basic or less or unknown, 2 = Upper secondary, 3 = Lower tertiary, and 4 = Higher tertiary) [31]. Income information is based on state taxation from the same period. Taxable incomes were first log-transformed, and the data were standardised separately at each data collection point by sex. Participants who were recorded as retired at a specific data collection point were not included in the standardisation at that point. Maximum income level during adulthood was defined by using standardised values and split into thirds (1 = lowest, 2 = intermediate, 3 = highest).

In total, 12304 men and women had information on their marital status at 5 year intervals between 1970 and 2000. We split these into two groups: ever married (entry “married”, “divorced” or “widowed” at any of the time points)/never married (none of these entries at any time point) and ever divorced (entry “divorced” at any of the time points)/never divorced (entry “single”, “married” or “widowed” at any time points but no entry “divorced”).

### Analysis

An independent-samples t-test was conducted to compare the birth characteristics for people born to unmarried mothers and people born married mothers. The associations between mother’s marital status and offspring’s SEP and marital status in adulthood were analysed using multinomial logistic regression analysis with three models. The first model included year of birth and sex). The second included, in addition, perinatal and childhood factors associated with being born to an unmarried mother: birth weight, birth order, mother’s age, length of gestation and evacuation abroad without parents during World War II [25, 28]. While these factors could strictly be considered to be on the causal pathway between being born to an unmarried mother and the outcomes, we include these in this and consequent models to illustrate to what extent the associations with being born to an unmarried mother are independent of these perinatal and childhood factors. Associations of these variables with outcomes are presented in Table 2. The third model also included mother’s occupational information as another indicator of childhood SEP. We also tested if any of these associations were moderated by sex by including an interaction term ‘mother’s marital status in childhood * sex’ in the regression. In addition, we tested associations between different types of families (by mother’s marital status and the presence and occupational status of the male caregiver) and adulthood outcomes. These analyses were controlled for the same variables as in the second model and also included a dummy variable representing mother’s marital status at birth and male caregiver’s highest occupational status during childhood. We also present these models controlled for mother’s occupation.

**Table 2:** Associations of covariates with adult socioeconomic position: ORs and 95 % CIs

Educational attainment in adulthood				
	Upper tertiary	Lower tertiary	Upper secondary	Basic or less or unknown
Year or birth	Ref.	.98 (.96–1.00)	1.01 (.99–1.03)	.94 (.92–.96)
Sex (female vs. male)	Ref.	1.56 (1.36–1.78)	1.43 (1.25–1.63)	1.85 (1.63–2.10)
Birth order (later vs. firstborn)	Ref.	1.24 (1.08–1.42)	1.37 (1.20–1.57)	1.75 (1.55–1.98)
Birth weight (kg)	Ref.	1.04 (.90–1.19)	.92 (.80–1.06)	.91 (.80–1.04)
Mother’s age	Ref.	.99 (.98–1.00)	.97 (.96–.99)	.99 (.97–1.00)
Length of gestation (weeks)	Ref.	1.00 (.96–1.03)	.99 (.96–1.03)	1.01 (.98–1.04)
Evacuation abroad without parents during World War II	Ref.	1.27 (1.00–1.62)	1.70 (1.35–2.15)	1.84 (1.47–2.29)
Mother’s occupation (clerical vs. manual worker)	Ref.	.54 (.46–.62)	.25 (.21–.29)	.23 (.20–.26)
Occupational status in adulthood				
	High official	Low official	Self-employed	Manual worker
Year or birth	Ref.	1.03 (1.01–1.06)	1.03 (1.00–1.06)	1.04 (1.02–1.06)
Sex (female vs. male)	Ref.	3.65 (3.23–4.13)	1.48 (1.27–1.73)	.97 (.86–1.09)
Birth order (later vs. firstborn)	Ref.	1.12 (.99–1.26)	1.27 (1.09–1.48)	1.43 (1.27–1.61)
Birth weight (kg)	Ref.	.98 (.86–1.11)	1.01 (.93–1.28)	.88 (.78–1.00)
Mother’s age	Ref.	1.00 (.98–1.01)	.99 (.98–1.01)	.99 (.98–1.00)
Length of gestation (weeks)	Ref.	.99 (.96–1.03)	1.02 (.98–1.06)	1.01 (.98–1.04)
Evacuation abroad without parents during World War II	Ref.	1.51 (1.23–1.85)	1.36 (1.05–1.76)	1.79 (1.47–2.18)
Mother’s occupation (clerical vs. manual worker)	Ref.	.50 (.44–.57)	.58 (.49–.68)	.24 (.21–.28)
Income in adulthood				
	Highest third	Intermediate third		Lowest third
Year or birth	Ref.	1.08 (1.06–1.09)		.96 (.95–.98)
Sex (female vs. male)	Ref.	.93 (.86–1.02)		.99 (.91–1.08)
Birth order (later vs. firstborn)	Ref.	1.14 (1.04–1.24)		1.30 (1.19–1.42)
Birth weight (kg)	Ref.	.83 (.76–.91)		.81 (.74–.89)
Mother’s age	Ref.	1.00 (.99–1.01)		1.00 (.99–1.01)
Length of gestation (weeks)	Ref.	1.01 (.98–1.03)		.98 (.96–1.01)
Evacuation abroad without parents during World War II	Ref.	1.09 (.94–1.26)		1.25 (1.09–1.43)
Mother’s occupation (clerical vs. manual worker)	Ref.	.57 (.52–.63)		.45 (.41–.50)

All models are adjusted for year of birth and sex

*OR* odds ratio, *CI* confidence intervals

## Results

Maternal and neonatal characteristics are shown in Table 1. Unmarried mothers were on average younger, shorter and had a lower body mass index in late pregnancy. They also had a lower occupational status than married women. Their offspring were on average lighter and shorter than children born to married mothers, they were born at an earlier gestational age and were more often firstborn. In families of unmarried mothers who later had a male caregiver, the male caregiver had on average a lower occupational status than male caregivers in families of married mothers. This difference was less strong than the difference in occupational status between unmarried and married mothers.

Table 3 presents odds ratios (ORs) and 95 % confidence intervals (CIs) from the multinomial logistic regression predicting the highest educational attainment, occupational status and maximum income in adulthood according to mother’s marital status at the time of birth. Compared to children of married mothers, children of unmarried mothers were more likely to have lower educational and occupational levels and income as adults. The associations were graded, so that the ORs increased with decreasing educational, occupational and income categories. They were little changed after adjusting for year of birth, sex, birth order, mother’s age, length of gestation and wartime evacuation status. Further adjustments with mother’s height and body mass index in late pregnancy attenuated the associations slightly (data not shown). In addition, after adjusting for mother’s occupational status the associations were somewhat attenuated, although most remained statistically significant. All associations were similar in men and women (p-values for interaction sex * unmarried mother >0.05).

**Table 3:** ORs and 95 % CIs for adulthood SEP according to mother’s marital status at birth

Educational attainment in adulthood (*n* = 12304)				
	Upper tertiary	Lower tertiary	Upper secondary	Basic or less or unknown
Number of mothers unmarried/total	25/1300	87/2726	162/3060	293/5218
Model 1	Ref.	1.67	2.87	2.95
		(1.07–2.62)	(1.87–4.40)	(1.95–4.47)
Model 2	Ref.	1.71	3.04	3.40
		(1.08–2.73)	(1.96–4.72)	(2.17–5.20)
Model 3	Ref.	1.53	2.28	2.48
		(.98–2.43)	(1.46–3.56)	(1.61–3.83)
Occupational status in adulthood (*n* = 12047)				
	High official	Low official	Self–employed	Manual worker
Number of mothers unmarried/total	36/1527	172/4468	55/1191	278/4861
Model 1	Ref.	1.62	2.02	2.58
		(1.21–2.34)	(1.32–3.09)	(1.81–3.66)
Model 2	Ref.	1.55	2.16	2.75
		(1.06–2.57)	(1.39–3.35)	(1.92–3.95)
Model 3	Ref.	2.29	1.86	2.0
		(.88–1.89)	(1.21–2.93)	(1.38–2.89)
Income in adulthood (*n* = 12251)				
	Highest third	Intermediate third	Lowest third	
Number of mothers unmarried/total	130/4084	189/4081	246/4086	
Model 1	Ref.	1.53	1.91	
		(1.22–1.93)	(1.54–2.37)	
Model 2	Ref.	1.63	2.09	
		(1.28–2.07)	(1.66–2.64)	
Model 3	Ref.	1.43	1.75	
		(1.13–1.83)	(1.39–2.21)	

Educational attainment and occupational status indicate maximum position as recorded in the National Census at 5-year intervals between 1970 and 2000. Maximum income level is based on state taxation from the same period. For each recorded year, taxable incomes were log transformed and standardised separately for men and women

Model 1 adjusted for year of birth and sex

Model 2 adjusted as in model 1 + birth order, birth weight, mother’s age, length of gestation and evacuation abroad without parents during World War II

Model 3 adjusted as in model 2 + mother’s occupational status (clerical/worker)

*OR* odds ratio, *CI* confidence intervals, *SEP* socioeconomic position (education, occupation and income)

We then compared different types of families based on mother’s marital status at birth and later presence of a male caregiver and his occupational status. As a reference group we used children who were born to married mothers and whose fathers/male caregivers were manual workers. Compared with these children, children born to an unmarried mother who had no male caregiver during childhood had the highest chance of ending up in the lower SEP groups in adulthood (Tables 4, 5 and 6). Also children born to unmarried mothers who later had a manual worker male caregiver were more likely to attain a lower adult SEP than corresponding children born to married mothers. The tables show that children who had an unmarried mother and who later had a male caregiver in a middle class profession had similar SEP prospects as the reference group of children of married mothers and manual worker fathers. However, these children attained a lower SEP than children of married mothers whose fathers had a middle class profession. After adjusting for mother’s occupational status the ORs attenuated a little). In other words, having a mother in a middle class profession, regardless of whether the child was born to a married or unmarried mother, seemed to increase the possibility of reaching a higher SEP in adulthood.

**Table 4:** ORs and 95 % CIs for adulthood education according to mother’s marital status and parental occupation

Educational attainment in adulthood (*n* = 12304)								
	Upper tertiary		Lower tertiary		Upper secondary		Basic or less or unknown	
	Model 1	Model 2	Model 1	Model 2	Model 1	Model 2	Model 1	Model 2
Groups defined by mother’s marital status, the presence of male caregiver, and his occupation								
Married mother, male caregiver worker	Ref.	Ref.	Ref.	Ref.	Ref.	Ref.	Ref.	Ref.
Unmarried mother, no indication of male caregiver^a^	Ref.	Ref.	2.01	1.76	2.86	2.14	3.32	2.43
			(.89–4.57)	(.77–4.00)	(1.30–6.27)	(.97–4.72)	(1.54–7.16)	(1.12–5.27)
Unmarried mother, male caregiver worker^b^	Ref.	Ref.	1.65	1.33	2.49	1.62	2.50	1.58
			(.67–4.06)	(.54–.30)	(1.06–5.85)	(.69–3.84)	(1.08–5.77)	(.68–3.66)
Unmarried mother, male caregiver middle class^c^	Ref.	Ref.	.71	.67	.64	.57	0.60	.52
			(.34–1.50)	(.32–1.42)	(.31–1.32)	(.27–1.17)	(.30–1.18)	(.26–1.03)
Married mother, male caregiver middle class	Ref.	Ref.	.63	.63	.30	.32	.25	.26
			(.55–.72)	(.54–.75)	(.26–.34)	(.27–.37)	(.22–.30)	(.23–.31)
Mother’s occupational status								
Mother worker		Ref.		Ref.		Ref.		Ref.
Mother middle class		Ref.		.65		.39		.38
				(.55–.76)		(.33–.46)		(.32–.44)

Maximum educational attainment as recorded in the National Census at 5-year intervals between 1970 and 2000

Model 1 adjusted for year of birth, sex, birth order, birth weight, mother’s age, length of gestation, evacuation abroad without parents during World War II, mother’s marital status (married/unmarried) and male caregiver’s highest attained occupational status during childhood (missing, worker or clerical)

Model 2 adjusted as in model 1 + mother’s occupational status

*OR* odds ratio, *CI* confidence intervals

^a^The mothers (*n* = 303) who have no indication of a male caregiver at any time point

^b-c^The mothers (*n* = 334) who were unmarried at the time of childbirth but the family had a male caregiver later on, according to the child welfare clinic or school records

**Table 5:** ORs and 95 % CIs for adulthood occupation according to mother’s marital status and parental occupation

Occupational status in adulthood (*n* = 12047)								
	High official		Low official		Self-employed		Laborer	
	Model 1	Model 2	Model 1	Model 2	Model 1	Model 2	Model 1	Model 2
Groups defined by mother’s marital status, the presence of male caregiver, and his occupation								
Married mother, male caregiver worker	Ref.	Ref.	Ref.	Ref.	Ref.	Ref.	Ref.	Ref.
Unmarried mother, no indication of male caregiver^a^	Ref.	Ref.	2.03	1.68	2.78	2.45	2.90	2.20
			(1.04–4.40)	(.86–3.30)	(1.32–5.89)	(1.15–5.20)	(1.51–5.57)	(1.15–4.29)
Unmarried mother, male caregiver worker^b^	Ref.	Ref.	1.32	1.02	1.80	1.48	2.11	1.40
			(.66–2.65)	(.51–2.05)	(.80–4.05)	(.65–3.34)	(1.09–4.08)	(.72–2.72)
Unmarried mother, male caregiver middle class^c^	Ref.	Ref.	.41	.36	1.08	1.00	.56	.49
			(.21–.80)	(.19–.71)	(.52–2.27)	(.48–2.11)	(.30–.1.02)	(.26–.90)
Married mother, male caregiver middle class	Ref.	Ref.	.52	.51	.68	.70	.29	.32
			(.46–.59)	(.44–.59)	(.58–.80)	(.58–.84)	(.25–.32)	(.27–.37)
Mother’s occupational status								
Mother worker		Ref.		Ref.		Ref.		Ref.
Mother middle class		Ref.		.64		.68		.37
				(.56–.74)		(.57–.82)		(.32–.43)

Maximum occupational status as recorded in the National Census at 5-year intervals between 1970 and 2000

Model 1 adjusted for year of birth, sex, birth order, birth weight, mother’s age, length of gestation, evacuation abroad without parents during World War II, mother’s marital status (married/unmarried) and male caregiver’s highest attained occupational status during childhood (missing, worker or clerical)

Model 2 adjusted as in model 1 + mother’s occupational status

*OR* odds ratio, *CI* confidence intervals

^a^The mothers (*n* = 303) who have no indication of a male caregiver at any time point

^b-c^The mothers (*n* = 334) who were unmarried at the time of childbirth but the family had a male caregiver later on, according to the child welfare clinic or school records

**Table 6:** Adulthood income level in thirds according to mother’s marital status and parental occupation

Income level in adulthood in thirds (*n* = 12251)						
	Highest		Intermediate		Lowest	
	Model 1	Model 2	Model 1	Model 2	Model 1	Model 2
Groups defined by mother’s marital status, the presence of male caregiver, and his occupation						
Married mother, male caregiver worker	Ref.	Ref.	Ref.	Ref.	Ref.	Ref.
Unmarried mother, no indication of male caregiver^a^	Ref.	Ref.	1.59	1.44	2.20	1.94
			(1.11–2.28)	(1.00–2.06)	(1.56–3.09)	(1.37–2.74)
Unmarried mother, male caregiver worker^b^	Ref.	Ref.	1.27	1.09	1.47	1.21
			(.85–1.92)	(.73–1.65)	(.99–2.18)	(.81–1.79)
Unmarried mother, male caregiver middle class^c^	Ref.	Ref.	.99	.95	.93	.88
			(.60–.1.64)	(.58–.1.57)	(.56–1.52)	(.54–1.46)
Married mother, male caregiver middle class	Ref.	Ref.	.60	.63	.51	.56
			(.55–.66)	(.56–.70)	(.46–.56)	(.51–.63)
Mother’s occupational status						
Mother worker		Ref.		Ref.		Ref.
Mother middle class		Ref.		.70		.57
				(.64–.78)		(.52–.64)

Maximum income level in adulthood is based on state taxation as recorded in the National Census at 5-year intervals between 1970 and 2000. For each recorded year, taxable incomes were log transformed and standardised separately for men and women

Model 1 adjusted for year of birth, sex, birth order, birth weight, mother’s age, length of gestation, evacuation abroad without parents during World War II, mother’s marital status (married/unmarried) and male caregiver’s highest attained occupational status during childhood (missing, worker or clerical)

Model 2 adjusted as in model 1 + mother’s occupational status

*OR* odds ratio, *CI* confidence intervals

^a^The mothers (*n* = 303) who have no indication of a male caregiver at any time point

^b-c^The mothers (*n* = 334) who were unmarried at the time of childbirth but the family had a male caregiver later on, according to the child welfare clinic or school records

Children born to unmarried mothers were more likely to stay unmarried in adulthood compared with children of married mothers (Table 7). Again, the group with the highest odds comprised children born to an unmarried mother with no male caregiver during childhood. In analyses related to marital status in adulthood, we found no difference between any other family groups (Table 8). Children of unmarried mothers who ended up married in adulthood had a similar chance of divorce than those of married mothers.

**Table 7:** ORs and 95 % CIs for marital status in adulthood according to mother's marital status and parental occupation

	Married		Divorced	
	Never Married	Ever married	Never divorced	Ever divorced
Model 1	Ref.	.67 (.53–.86)	Ref.	1.20 (1.00–1.44)
	*n* = 1268	*n* = 11036	*n* = 8697	*n* = 3607
Model 2	Ref.	.66 (.51–.85)	Ref.	1.24 (1.02–1.49)
	*n* = 1210	*n* = 10476	*n* = 8257	*n* = 3429
Model 3	Ref.	.68 (.53–.89)	Ref.	1.20 (.99–1.49)
	*n* = 1210	*n* = 10476	*n* = 7337	*n* = 3074

Marital status as recorded in the National Census at 5-year intervals between 1970 and 2000

Model 1 adjusted for year of birth and sex

Model 2 adjusted as in model 1 + birth order, birth weight, mother’s age, length of gestation and evacuation abroad without parents during World War II

Model 3 adjusted as in model 2 + mother’s occupational status (clerical/worker)

*OR* odds ratio, *CI* confidence intervals

**Table 8:** ORs and 95 % CIs for adulthood marital status according to male caregiver’s SEP and mother’s marital status

	Married			Divorced		
	Never married	Ever married		Never divorced	Ever divorced	
		Model 1	Model 2		Model 1	Model 2
Groups defined by mother’s marital status, the presence of male caregiver, and his occupation						
Married mother, male caregiver worker	Ref.	Ref.	Ref.	Ref.	Ref.	Ref.
Unmarried mother, no indication of male caregiver^a^	Ref.	.61 (.43–.86)	.63 (.44–.89)	Ref.	1.21 (.93–1.56)	1.19 (.91–1.55)
Unmarried mother, male caregiver worker^b^	Ref.	.74 (.47–1.15)	.77 (.49–1.21)	Ref.	1.26 (.91–1.74)	1.22 (.88–1.69)
Unmarried mother, male caregiver middle class^c^	Ref.	.75 (.41–1.35)	.76 (.42–1.37)	Ref.	1.12 (.73–1.73)	1.12 (.73–1.73)
Married mother, male caregiver middle class		1.07 (.94–1.22)	1.06 (.92–1.22)	Ref.	.95 (.88–1.04)	.98 (.89–1.08)
Mother’s occupational status						
Mother worker	Ref.		Ref.	Ref.		
Mother middle class	Ref.		1.11 (.96–1.27)	Ref.		.89 (.81–.97)

Marital status as recorded in the National Census at 5-year intervals between 1970 and 2000. All analyses were controlled for year of birth, sex, birth order, birth weight, mother’s age, length of gestation, evacuation abroad without parents during World War II, mother’s marital status (married/unmarried) and male caregiver’s highest attained occupational status during childhood (missing, worker or clerical)

Model 1 adjusted for year of birth, sex, birth order, birth weight, mother’s age, length of gestation, evacuation abroad without parents during World War II, mother’s marital status (married/unmarried) and male caregiver’s highest attained occupational status during childhood (missing, worker or clerical)

Model 2 adjusted as in model 1 + mother’s occupational status

*OR* odds ratio, *CI* confidence intervals

^a^The mothers (*n* = 303) who have no indication of a male caregiver at any time point

^b-c^The mothers (*n* = 334) who were unmarried at the time of childbirth but the family had a male caregiver later on, according to the child welfare clinic or school records

## Discussion

We examined how mother’s marital status when giving birth was associated with offspring’s lifetime SEP and marital status. Our results showed that children born out of wedlock almost 80 years ago attained a lower SEP in adulthood and were more likely to remain unmarried than children of married mothers. The most disadvantaged group consisted of children born to an unmarried mother who did not have a male caregiver during childhood years. They were most likely to end up in lower SEP and to stay unmarried. Also children who were born to an unmarried mother and who later had a male caregiver attained a lower SEP than those born to married mothers. Furthermore, having a mother with a middle class profession increased the offspring’s probability of reaching a higher SEP in adulthood independently of mother’s marital status at birth.

Previous studies have reported an association between single parent background and lower SEP in later life [12, 14, 16, 17] but there are also studies showing no association after childhood socioeconomic circumstances are accounted for [21, 23]. However, the majority of this previous research has focused on outcomes in early adulthood [12, 22, 32, 33] and treated divorced, separated, never married as well as widowed, as if they were a homogeneous single parent group [21, 33–35]. Fewer studies have focused only on children born out of wedlock and measured changes in SEP in later life [16, 17]. This kind of a study design has been used in a small number of studies from Sweden [16] and Denmark [17]. These studies have reported associations between out of wedlock birth and later life outcomes, including SEP as well as health, largely independent of childhood socioeconomic circumstances. Our results are in line with these studies. We found that out of wedlock birth was associated with worse SEP in adulthood according to all indicators. The associations we found, were not explained through socioeconomic circumstances in childhood even though mother’s occupation at birth, as a marker of childhood socioeconomic position, was positively related to the likelihood that a child would attain a higher SEP in adulthood. Previous Scandinavian studies have also reported that men, but not women, born out of wedlock tend to more often become widowers, experience divorce and stay unmarried themselves [15–17]. In the present study this association between out of wedlock birth and the tendency to stay unmarried was found among both sexes. However, we found no association between out of wedlock birth and experiencing of divorce.

Our study adds to this previous literature by showing that the long-term effects of single parent background remained, in both sexes, even when adulthood SEP was measured several times during adult life and was observed by using three different indicators of SEP.

Several mechanisms could explain these associations. First, being born to an unmarried mother may have meant a lack of material resources, for example food, during the foetal period and throughout childhood. Intrauterine growth, for which size at birth in relation to gestational age serves as a marker, has an important role for the development of vital organs such as the brain and may have long-term consequences for later development [36, 37]. Second, the social stigma experienced by children born out of wedlock may have lead to stress [11, 13] and have an impact on their upbringing, self-identity and which may subsequently translate to later life consequences including SEP. From an evolutionary perspective, investments made for a child can differ greatly between single parent and two parent families. In two parent families both parents usually have more resources, e.g. income, time and attention, while a single parent may have to focus much of the social and material resources on earning a livelihood and possibly combating social stigma [11, 12, 38, 39]. If a single mother later is married it may improve children’s standard of living although relationships in stepfamilies may be tense [11, 40].

The strong associations between mother’s marital status and offspring SEP in adulthood we found are likely to contribute to the association between low childhood SEP and health in later life. However, our data also show that many people born to an unmarried mother gain a good education and occupation despite early disadvantage. Moreover, it is important to highlight that in any study out of wedlock birth should be understood as an indicator of social disadvantage in the specific historical context of the study period. Our cohort members were born between 1934 and 1944 during an era when marriage was the norm. Therefore no direct analogy can be drawn to birth outside marriage and single parenting in contemporary societies. There are, however, still many contemporary contexts, where single mothers may have limited material and social resources similarly to participants of the present study.

Our results deserve an additional note from a technical perspective. Father’s occupational status is a widely used indicator of childhood SEP in epidemiological studies. It is important to realise that a missing father’s occupation may indicate a child born to an unmarried mother. In such cases it may be wiser to include such children as a separate category rather than to exclude them because of missing data, assess SES by the single parent’s occupation, or to use a purely statistical imputation technique. The limitations of the HBCS have been discussed [41]. Data on marital status, SEP and birth characteristics came from reliable records. While SEP in childhood was based only on occupational information, we have used both the mother’s and possible male caregiver’s occupations and classified occupational status based on modern standards of classifying historical occupational data. We have no data, however, on the remaining family members other than their number. Moreover, the adult occupational data are available from year 1970 onwards, when the cohort members were aged 26–36 years, precluding the assessment of socio-economic trajectories in adult life. We have no data on cohabitation without marriage. This is unlikely to be a major limitation for our main exposure, as marriage was the norm when the study participants were born, but constitutes a limitation for marital status as an outcome. Our study population may not be representative of all people living in Helsinki at that time. However, the distribution of occupational categories is consistent with the general occupational distribution in Helsinki 80 years ago among married HBCS families [42]. Child welfare clinic services were voluntary and free of charge and our data indicate that these clinics were attended by children from all socioeconomic groups. It is also possible that some unmarried mothers were recorded as married in the records and thus the married group may include a proportion of “false negatives”. This would be expected, if anything, to reduce the differences between children of married and unmarried mothers.

## Conclusion

This life course study shows that children born out of wedlock carry a socioeconomic disadvantage throughout life. As compared with children born to married mothers, they have approximately three-fold odds of ending up in the lowest than in the highest educational and occupational categories. Most likely to end up in these categories are children born to unmarried mothers who have no male caregiver during childhood. These associations are not explained by other socioeconomic factors as indicated by mother’s and possible male caregiver’s occupational statuses. This disadvantage starting in early life is likely to have a substantial effect on lifetime health.

## Abbreviations

CI: Confidence Interval
HBCS: the Helsinki Birth Cohort Study
OR: Odds Ratio
SEP: Socioeconomic Position
